# Cerium-coated Fe_3_O_4_ nanocomposite enhances salt stress tolerance in maize by modulating photosynthetic efficiency, antioxidant defense, and cellular ultrastructure

**DOI:** 10.3389/fpls.2026.1768765

**Published:** 2026-04-15

**Authors:** Abdul Salam, Muhammad Rehman, Zeya Deng, Shiqi Zhu, Xin Wang, Jinzhe Chang, Muhammad Zeeshan, Zhixiang Zhang, Chen Zhao

**Affiliations:** 1 State Key Laboratory of Green Pesticide, South China Agricultural University, Guangzhou, China; 2 Zhejiang Key Laboratory of Crop Germplasm, Department of Agronomy, College of Agriculture and Biotechnology, Zhejiang University, Hangzhou, China

**Keywords:** cerium, iron oxides, maize, nanocomposite, salt stress tolerance

## Abstract

**Introduction:**

Soil salinity, characterized by excessive soluble salts in the root zone, affects over 950 million hectares globally and continues to expand due to climate change, seawater intrusion, and unsustainable agricultural practices. High salinity imposes osmotic and ionic stress on plants, disrupting water and nutrient uptake, inducing ion imbalance, and triggering excessive reactive oxygen species (ROS) production, which leads to oxidative damage and impaired plant growth. Nanotechnology is an emerging strategy for enhancing plant stress tolerance, and combining nanomaterials with complementary properties offers a promising approach to improve plant resilience under such conditions.

**Methods:**

This study evaluated the efficacy of cerium-coated triiron tetraoxide (Fe_3_O_4_@Ce) nanocomposites (NCs) applied at different concentrations to mitigate salt stress in maize. Seedlings were subjected to 150 mM NaCl and treated with foliar-applied NCs. Growth parameters, photosynthesis, oxidative stress markers, ion homeostasis, and ultrastructural changes were analyzed.

**Results and Discussion:**

Salt stress significantly reduced plant growth, biomass, and photosynthetic efficiency while increasing oxidative damage and disrupting cellular ultrastructure. In contrast, NC application enhanced biomass production, chlorophyll content, antioxidant enzyme activities, and osmolyte accumulation, while reducing malondialdehyde (MDA), superoxide (O_2_•^⁻^), and hydrogen peroxide (H_2_O_2_) levels. The treatment decreased Na^+^ accumulation and increased K, Ca, and Mg uptake, improving ionic homeostasis and alleviating oxidative stress. Gas exchange parameters and stomatal structure were restored, and ultrastructural analyses confirmed recovery of mesophyll and root cell integrity. These results indicate that Fe_3_O_4_@Ce nanocomposites mitigate salt stress through coordinated regulation of antioxidant defense, ion balance, and cellular structure, highlighting their potential as an eco-friendly strategy for enhancing crop resilience.

## 1 Introduction

Soils containing excessive soluble salts or high levels of exchangeable sodium in the root zone are classified as soil salinity (Chhabra, 2021). Salinity currently affects more than 950 million hectares globally, with this area expanding at an alarming rate due to both natural processes and anthropogenic activities (Yang and Guo, 2018). Climate change, seawater intrusion, over-irrigation, the use of saline water, and excessive fertilizer application have all contributed to soil salinity (Qian et al., 2021; Zhang et al., 2016). High level of salt in the soil imposes osmotic and ionic stress on plants, thereby impairing water and nutrient uptake (Negrão et al., 2017). Initially, salt stress reduces the water potential in the rhizosphere, restricting water uptake by plant roots. This is followed by the accumulation of toxic ions, primarily sodium (Na^+^) and chloride (Cl^−^), which interfere with ion homeostasis and nutrient uptake, especially of essential elements such as potassium (K^+^) and calcium (Ca^+^) (Zhang et al., 2017). The resulting nutrient imbalance disrupts metabolic processes and slows plant growth. In addition, both osmotic and ionic stresses trigger excessive production of reactive oxygen species (ROS). These ROS can damage DNA, lipids, proteins, and cellular membranes, ultimately causing oxidative stress and impairing normal cellular functions (Liu et al., 2021).

Salt stress affects plants throughout their entire life cycle, from seed germination to flowering and grain filling. It leads to reduced photosynthetic efficiency, disrupted membrane function, hormonal imbalances, and nutrient deficiencies, which collectively result in decreased plant growth and yield (Parihar and Bora, 2019; Wu et al., 2018). Soil salinization alters the physicochemical properties of the soil, further exacerbating nutrient imbalances and limiting the efficiency of plant nutrient uptake mechanisms (Yang and Guo, 2018). Under saline conditions, altered soil pH and ion competition can reduce the solubility and availability of Fe, leading to deficiencies that further impair plant health (Vélez-Bermúdez and Schmidt, 2023). Conventional approaches to augment salt tolerance in crops include the application of chemical growth regulators, osmoprotectants, and inorganic nutrients. For instance, boron supplementation can improve ion balance and stress tolerance (Ehsan-Ul-Haq et al., 2009), however, its narrow therapeutic window makes it prone to toxicity at slightly elevated concentrations (Karthika et al., 2018). Similarly, conventional iron fertilizers often exhibit poor solubility and bioavailability in high-pH, saline soils, which limits their effectiveness (Zia-ur-Rehman et al., 2023). While these methods have shown some success in improving plant tolerance to salinity, their long-term sustainability and environmental safety remain concerns (Rahman and Zhang, 2018).

In recent years, advances in nanotechnology have opened new opportunities to mitigate the adverse effects of salinity on plants, offering a sustainable approach for salinity stress management (Singh et al., 2025). The application of nanoparticles (NPs) enhances seed germination, improving nutrient uptake, regulating plant growth, and boosting tolerance to various biotic and abiotic stresses (El-Mogy et al., 2025; Kah and Kookana, 2020). For example, silica (SiO_2_) NPs can regulate ion transporters and reduce Na^+^ accumulation in leaves (Liu et al., 2021). Zinc oxide NPs (ZnO) enhanced photosynthesis by boosting electron transport, PSII quantum yield, proton conductance, and mineral levels, resulting in improved plant health (Mahawar et al., 2024). Selenium (Se) NPs combined with ZnO enhanced plant zinc levels and brought physiological and biochemical parameters close to normal, resulting in increases of 46.32% in plant height, 70.53% in root length, and 100.7% in grains per spike under salinity stress (Mishra et al., 2025). These nanomaterials not only improve plant performance under stress but also minimize environmental risks associated with conventional agrochemicals (Kah and Kookana, 2020). Similarly, Iron oxide (FeO) NPs have been shown to enhance iron uptake in saline soils (Zia-ur-Rehman et al., 2023), while cerium oxide (CeO_2_) NPs act as ROS scavengers, mimicking endogenous antioxidant enzymes under salt (Khan et al., 2021). However, the combined use of these materials in the form of a nanocomposite has not yet been reported. Therefore, the present study investigates the potential of cerium-coated Fe_3_O_4_ nanocomposites, which are expected to simultaneously improve nutrient availability and reduce oxidative damage, thereby enhancing plant tolerance to salinity. Unlike single-component iron or cerium NPs cerium-coated Fe_3_O_4_ NCs integrate the magnetic and nutritional properties of Fe_3_O_4_ with the redox-active and antioxidant functions of cerium, enabling more efficient regulation of ROS and improved stress tolerance under saline conditions. Therefore, the present study aimed to develop iron–cerium NCs to harness the complementary benefits of both components and enhance plant tolerance to salt stress.

Maize (*Zea mays* L.) is one of the most important cereal crops globally, valued for its high yield potential, versatility, and economic importance (Farooq et al., 2023). It serves as a key raw material for food, feed, and industrial applications. Despite its adaptability to various agro-climatic conditions, maize is moderately sensitive to salinity stress, which significantly affects germination, growth, and yield (Yu et al., 2023). With the increasing impact of global climate change and abiotic stresses, developing effective and sustainable strategies to enhance maize tolerance has become a pressing research priority (Faheem Jan et al., 2025; Salam et al., 2025b).

## 2 Methods and materials

### 2.1 Materials

The chemicals, including cerium nitrate hexahydrate [Ce(NO_3_)_3_·6H_2_O], ammonia solution (NH_3_·H_2_O), iron(ii) chloride tetrahydrate (FeCl_2_·4H_2_O), and iron(iii) chloride hexahydrate (FeCl_3_·6H_2_O), were acquired from Shanghai Macklin Bio-Technology Co., Ltd. (Shanghai, China). Polyvinyl pyrrolidone (PVP), ethanol, and other reagents used for plant biochemical analyses were obtained from Shanghai Aladdin Bio-Technology Co., Ltd. (Shanghai, China). All chemicals used were of analytical grade and employed without further refinement or purification. Deionized water was obtained from the laboratory’s ultrapure water system, and other reagents were used as received.

### 2.2 Preparation of iron cerium nanocomposite

Fe_3_O_4_@Ce NCs were synthesized using ferric (FeCl_3_·6H_2_O), ferrous (FeCl_2_·4H_2_O), and cerium [Ce(NO_3_)_3_·6H_2_O] salts as precursors. Briefly, FeCl_2_·4H_2_O (15 mM) and FeCl_3_·6H_2_O (30 mM) were dissolved in a Milli-Q water-ethanol mixture (4:1, v/v) at a 1:2 molar ratio under magnetic stirring. The solution was heated to 80 °C, and the pH was adjusted to 11 with NaOH, followed by continuous stirring for 1 h. PVP was added during co-precipitation as a capping agent. The NPs were magnetically separated, washed repeatedly with deionized water and ethanol, and stored in ethanol.

For cerium coating, Fe_3_O_4_ NPs (1 g) were dispersed in 50 mL of ethanol and subjected to sonication for 30 min to obtain a stable ferrofluid. A 4.5 mM [Ce(NO_3_)_3_·6H_2_O] solution in Milli-Q water ethanol (4:1, v/v) was added dropwise to the dispersion under continuous stirring at room temperature for 1 h. The resulting grey Fe_3_O_4_@Ce suspension was aged for 24 h, magnetically separated, and repeatedly washed with deionized water and ethanol until free of anionic impurities. The purified product was oven-dried at 80 °C for 12 h and calcined at 500 °C for 5 h to enhance crystallinity and phase purity.

### 2.3 Characterization of Fe_3_O_4_@Ce NCs

The morphology and microstructure of Fe_3_O_4_@Ce NCs were characterized by scanning electron microscopy (SEM, Hitachi SU8010, Japan) with elemental composition verified by energy-dispersive spectroscopy (EDS), and transmission electron microscopy (TEM, FEI Tecnai G2 F20). The surface functional groups were analyzed using Fourier-transform infrared spectroscopy (FTIR, Bruker Vertex 70), and crystalline phases were characterized by X-ray diffraction (XRD, Rigaku MiniFlex600, Japan).

### 2.4 Plant cultivation and treatment application

Maize seeds (*Zea mays* L., cultivar Yunrui 62) were obtained from the Yunnan Academy of Agricultural Sciences, China. For germination purposes, the seeds were initially surface-sterilized with 5% sodium hypochlorite for 10 min, immersed in 70% ethanol for 1 min, and then washed five times with double-distilled water (ddH_2_O). Sterile seeds were placed on Petri dishes lined with moistened filter paper and kept in the dark for 3 days to initiate germination. Germinated seedlings were then shifted to pots (4 plants/pot) containing 2 kg of soil and maintained under greenhouse conditions. Each pot was initially irrigated with 100 mL of distilled water to maintain approximately 80% soil moisture. Salinity stress was induced on day 11 after sowing by applying 150 mM NaCl. Salt concentration of 150 mM NaCl was selected based on previous studies in maize (Li A. et al., 2025; Zhang S. et al., 2025), where this level is commonly used to induce significant physiological and molecular stress responses while maintaining seedling viability. On the following day, foliar treatments of Fe_3_O_4_@Ce NCs were applied at three concentrations (25, 50, and 100 mg L^−1^). During the stress period, pots received 150 mM NaCl at 3-day intervals without additional watering. Control plants were irrigated with distilled water and received no treatment. Greenhouse conditions were adjusted to 28 °C, 70% relative humidity, and a 10–14 h photoperiod (600 µmol m^−^² s^−^¹ light intensity). Environmental parameters were monitored and adjusted to ensure uniform growth conditions.

### 2.5 Measurement of growth attribute

After 13 days of salt treatment, the plants were carefully uprooted, washed to remove soil, and their shoot and root length measured with a centimeter-scale ruler. Samples were washed with ddH_2_O, blotted dry with filter paper, and weighed to determine fresh biomass. The samples were then oven-dried at 70 °C for 72 h, and dry biomass was recorded using an electronic balance.

### 2.6 Quantification of chlorophyll content and photosynthetic attributes

The chlorophyll contents (chl a, chl b) and carotenoids were quantified from 0.1 g fresh leaf tissue in 5 mL 80% (v/v) acetone, centrifuged at 5000 × g for 10 min, and quantified by absorbance at 663, 645, 450, and 470 nm using a UV spectrophotometer (UV-2600, Shimadzu, Japan). Relative chlorophyll content was assessed on the second fully expanded leaf using a SPAD meter (SPAD-502, Minolta, Japan). Net photosynthetic rate (*Pn*) and stomatal conductance (*gs*) were measured on the same leaf between 10:00 and 12:00 h with a portable photosynthesis system (LI-6400XT, LI-COR, USA).

### 2.7 Quantification of antioxidant enzyme activity

Fresh root and shoot tissues (0.3 g) were ground to a fine powder in a pre-chilled mortar and pestle and homogenized in 6 mL of ice-cold phosphate buffer (50 mM, pH 7.8). The homogenates were centrifuged at 12,000 rpm for 15-20 min at 4 °C, and the supernatants were used for enzyme assays. To evaluate antioxidant enzyme activities, SOD, POD, APX, and CAT were measured following the protocol described by Zhang et al. (2008).

### 2.8 Determination of malondialdehyde and reduced glutathione contents

Leaf and root tissues (0.5 g) were homogenized in 5% metaphosphoric acid containing 1 mM EDTA and centrifuged at 12,000 g for 10 min at 4 °C. The supernatant was used to quantify reduced glutathione (GSH) content following the method of Hasanuzzaman et al. (2013). Lipid peroxidation was evaluated by determining MDA content using the thiobarbituric acid (TBA) reaction method as defined by Heath and Packer (1968).

### 2.9 Determination of hydrogen peroxide and superoxide anion

Hydrogen peroxide (H_2_O_2_) content was determined using a commercial kit (BC3590, Solarbio, China). Fresh leaf and root tissues (0.1 g) were ground under chilled conditions, extracted with 1 mL reagent, and centrifuged at 8000 × g for 10 min at 4 °C. Absorbance of the supernatant was read at 415 nm (UV-2600, Shimadzu, Japan), and concentrations were calculated from a standard curve. Superoxide anion (O_2_^•^−^^) was quantified using a detection kit (BC1295, Solarbio, China) by processing ground tissue in buffer with sequential centrifugation (12,000 × g and 10,000 × g), followed by absorbance measurement at 530 nm with a microplate reader, as reported previously Qi et al. (2023).

### 2.10 Quantification of osmolytes

To quantify soluble sugar, 0.5 g of fresh leaf tissue was homogenized in 10 mL of distilled water and then centrifuged at 8000 × g for 15 minutes at 4 °C. Mixed 1 mL of the supernatant with 4 mL of anthrone reagent and heated in a boiling water bath at 100 °C for 5 min. Once cooled to room temperature, absorbance was measured at 620 nm. The soluble sugar content was determined using a standard calibration curve. To measure soluble protein, fresh seedlings (0.1 g) were crushed in 1 mL of phosphate-buffered saline (PBS, pH 7.4), then centrifuged at 8000 × g for 10 minutes at 4 °C, and the supernatant was collected. A 5 µL aliquot was mixed with 250 µL of Coomassie Brilliant Blue G-250 reagent, and absorbance at 595 nm was measured after 30 min. Protein content was calculated using a standard curve (Salam et al., 2025a). Glycine betaine content was determined following Li and Zhu (2015). Briefly, 0.3 g of tissue was ground into powder and extracted in distilled water (200 rpm, 28 °C, 24 h). Extracts were centrifuged at 5000 × g for 15 min, pre-cooled (4 °C, 30 min), and reacted with Reinecke salt solution (pH 1) for 4 h at 4 °C to form red precipitates. The precipitates were rinsed three times with pre-cooled diethyl ether (99%), dissolved in 70% acetone, and adjusted to 10 mL. Absorbance was recorded at 525 nm, and glycine betaine levels were quantified via a standard calibration curve, expressed as µmol g^−^¹ fresh weight (FW). Proline content was assessed using the acid-ninhydrin method (Bates et al., 1973). Fresh leaf tissue (0.5 g) was extracted using sulfosalicylic acid, glacial acetic acid, and acidic ninhydrin, followed by heating at 100 °C. Following the reaction, 4 mL of toluene was added, and the absorbance of the chromophore-containing layer was measured at 520 nm using a Genesys UV–VIS spectrophotometer (PG Instruments Ltd., T80, UK). The results were reported as mg per gram of fresh weight (mg g^−^¹ FW).

### 2.11 Sample preparation for scanning electron microscopy analysis

Leaf samples from each seedling treatment were cut into small pieces (excluding veins) and subsequently fixed in 2.5% glutaraldehyde, prepared in 0.1 M phosphate-buffered saline (PBS, pH 7.0). The samples were post-fixed in 1% osmium tetroxide (OsO_4_) for 1–2 h, followed by three washes in PBS (15 min each). Dehydration was performed through a graded ethanol series (30%, 50%, 70%, 80%, 90%, 95%, and 100%), with each step lasting 15 min, and samples were washed twice in absolute ethanol (20 min each). Final dehydration was carried out using liquid CO_2_. Prepared specimens were examined and imaged using a scanning electron microscope (SEM; Hitachi SU-8010, Japan).

### 2.12 Sample preparation for transmission electron microscopy analysis

Leaf (excluding midribs) and root tissues were cut into small segments and fixed in 2.5% glutaraldehyde. Samples underwent three washes with 0.1 M phosphate buffer (pH 7.0) and post-fixed in 1% OsO_4_ for two h. Dehydration was performed through a graded ethanol series (30%, 50%, 70%, 80%, 90%, 95%, and 100%) for 15 min each, followed by two washes in absolute acetone for 20 min each. Specimens were infiltrated with a 1:1 mixture of Spurr resin and absolute acetone for 1 hour at room temperature, then with a 3:1 Spurr resin to acetone mixture for 3 hours. Samples were embedded in pure Spurr resin overnight and polymerized at 70 °C for 9 h. Ultrathin sections were prepared and examined using a transmission electron microscope (TEM; Hitachi H-7650, Japan) following the procedures of Azhar et al. (2022) and Salam et al. (2022).

### 2.13 Determination of sodium and other ion contents

Samples of dried roots and shoots were ground into a fine powder with a mechanical grinder. For metal quantification, 0.1 g of powdered tissue was digested in concentrated nitric acid (HNO_3_) for one h, followed by sequential heating at 120 °C and 140 °C for two h in a Dry Thermo Unit (Taitec, Tokyo, Japan). After complete digestion, the volume was adjusted with deionized water, and Na^+^, K^+^, Mg^2+^, and Ca^2+^ concentrations in leaf and root tissues were determined using inductively coupled plasma mass spectrometry (ICP-MS) (Rehman et al., 2025).

### 2.14 Gene expression analysis

Total RNA was isolated from shoot tissues using the TRIzol kit (B511311; Sangon, China) and subsequently reverse-transcribed in accordance with the manufacturer’s instructions. Maize actin gene served as the internal control for real-time quantitative PCR (qPCR). Primer sequences can be found in **Supplementary Table 1**. Relative gene expression levels were determined using the 2^−ΔΔ^Ct method (Livak and Schmittgen, 2001).

### 2.15 Statistical analysis

The experiments were conducted in 3 replicates per treatment and the data were analyzed with analysis of variance (ANOVA) in Statistix 8.1 (Analytical Software, USA). Results are presented as the mean ± standard deviation (SD). To identify significant differences among treatments, the Least Significant Difference (LSD) test was used at a significance level of p < 0.05. Treatment means were distinguished using alphabetical letters. Additionally, graphs were created with GraphPad Prism version 8.0 (GraphPad Software, USA).

## 3 Results and discussion

### 3.1 Characterization of iron cerium nanocomposite

**Figure 1:**
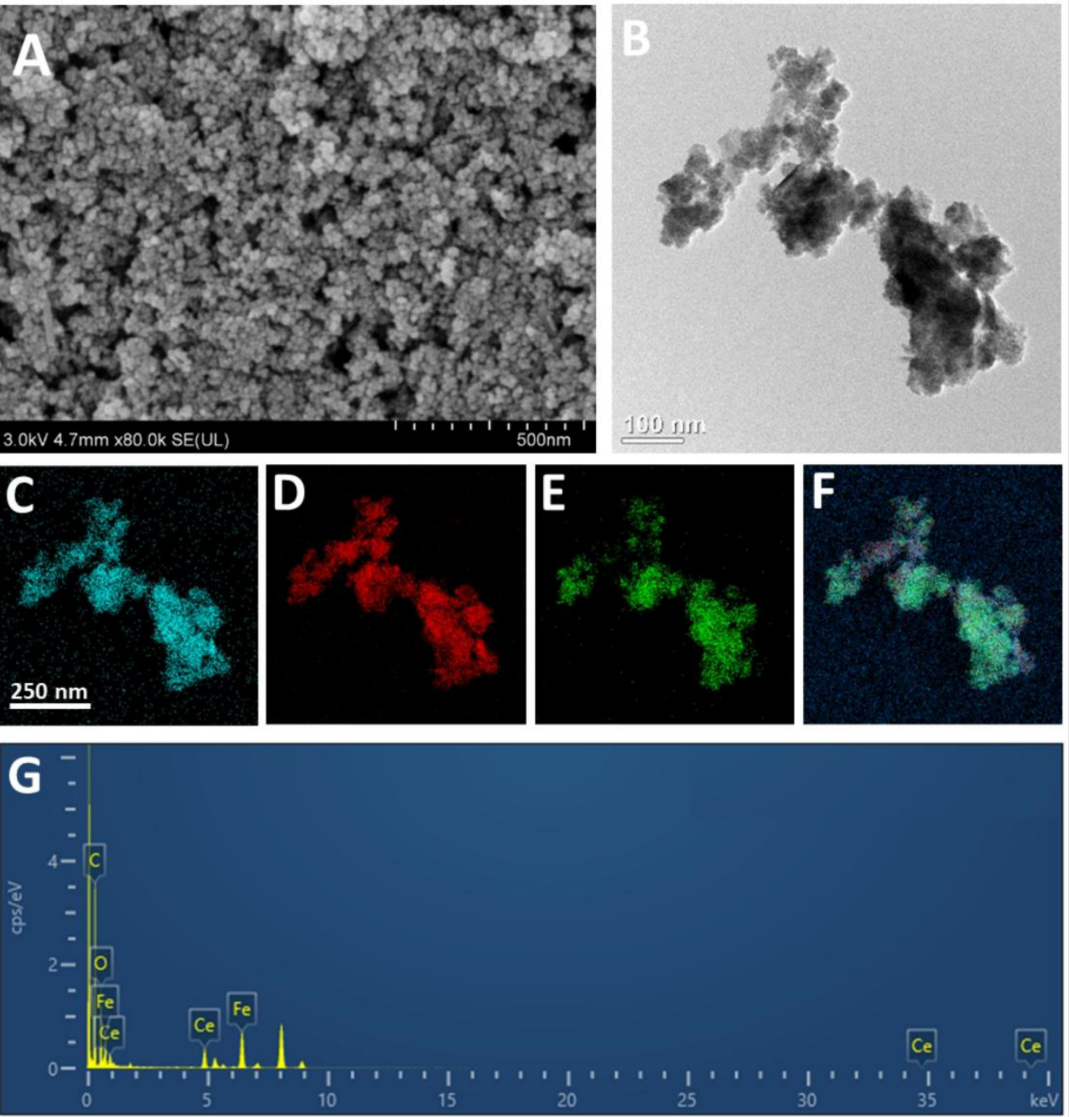
Characterization of Fe_3_O_4_@Ce NCs. **(A)** SEM image (scale bar = 500 nm); **(B)** TEM image (scale bar = 100 nm); **(C–G)** Elemental mapping by EDS (scale bar = 250 nm) showing the distribution of **(C)** oxygen, **(D)** iron, **(E)** cerium **(F)** merged mapping and **(G)** EDS spectra, confirming the elemental composition of the Fe_3_O_4_@Ce NCs.

The SEM analysis provided detailed insights into the nanostructure, particle size, and surface morphology of the cerium-coated iron oxide NCs (Fe_3_O_4_@Ce NCs) (**Figure 1A**). The micrographs revealed a relatively uniform and homogeneous distribution of NPs, exhibiting both spherical and slightly elongated morphologies with an average size of 22 nm. These observations suggest consistent particle formation and successful surface coating. TEM further validated the structural features and shape of the synthesized NCs, confirming their nanoscale dimensions and morphological consistency (**Figure 1B**). To determine the elemental composition and confirm the presence of constituent elements, Energy-Dispersive X-ray Spectroscopy (EDS) analysis was performed. The EDS spectra displayed clear and strong signals corresponding to cerium (Ce), iron (Fe), and oxygen (O), thereby confirming the successful synthesis and elemental incorporation of Ce onto the Fe_3_O_4_ nanostructures (**Figures 1C–G**). The XRD spectra showed high crystallinity of Fe_3_O_4_ with sharp diffraction peaks at 30.22°(220), 35.52°(311), 43.24°(400), 53.88°(422), 57.36°(511), 62.80°(440), 71.36°(620), 75.38°(533) respectively (**Supplementary Figures 1A, B**). These peaks are consistent with those reported in previous literature, confirming the successful synthesis of Fe_3_O_4_ (Irshad et al., 2024). The XRD analysis also revealed that Fe_3_O_4_@Ce NCs displayed diffraction peaks similar to those of Fe_3_O_4_ NPs, indicating that the Fe_3_O_4_ crystal structure was well preserved after Ce phase formation. The peaks of the Fe_3_O_4_@Ce NCs were broader than those of the Fe_3_O_4_ NPs, and their signal intensity was decreased due to the presence of the Ce coating (**Supplementary Figure 1B**). FTIR analysis revealed a strong absorption band at 587 cm^−^¹, characteristic of the Fe–O stretching vibration in Fe_3_O_4_ NPs (**Supplementary Figure 1C**). The peaks at 3377 cm^−^¹ and 1645 cm^−^¹ were assigned to O–H stretching and bending vibrations, respectively, arising from surface-adsorbed water molecules. Notably, the broad O–H band above 3000 cm^−^¹ became sharper after cerium coating, suggesting an increased number of surface hydroxyl groups on the Fe_3_O_4_@Ce NCs (Mohapatra et al., 2013). In Fe_3_O_4_@Ce, the additional band at 629 cm^−^¹, together with the 581 cm^−^¹ signal, represents intrinsic Fe–O–Ce stretching vibrations at the tetrahedral sites (Jouyandeh et al., 2019). The peak at 1538 cm^−^¹ is attributed to N–C stretching and N–H bending of –CO–NH– groups from residual surface ligands (Lakhotia et al., 2018). After cerium incorporation, the main Fe–O band shifted slightly from 581 to 587 cm^−^¹. This shift, along with peak broadening and reduced intensity, is consistent with cation vacancies in the Fe_3_O_4_ lattice caused by Ce substitution at sites previously occupied by Fe ions (Anushree et al., 2015; Qi et al., 2017).

### 3.2 Iron cerium NCs improved plant growth

**Figure 2:**
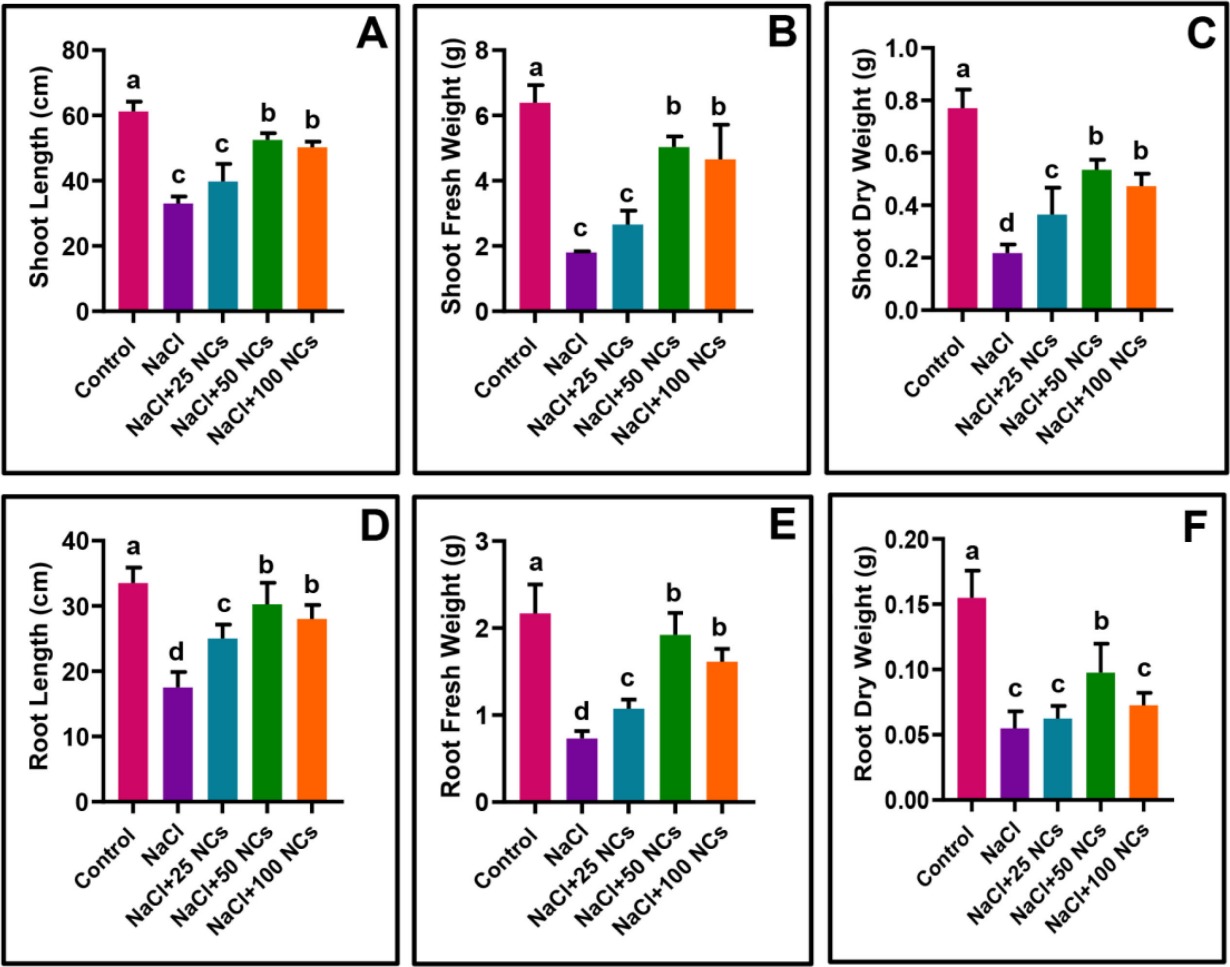
Effects of Fe_3_O_4_@Ce NCs on maize growth and biomass under salt stress: **(A)** Shoot length, **(B)** Shoot fresh weight, **(C)** Shoot dry weight, **(D)** Root length, **(E)** Root fresh weight, **(F)** Root dry weight at 25, 50, 100 mg L^−1^ concentration.

The results showed that salt stress significantly reduced the growth attributes including shoot length (SL) by 46.1%, shoot fresh weight (SFW) by 71.8%, shoot dry weight (SDW) by 71.75%, root length (RL) by 46.9%, root fresh weight (RFW) by 66.2%, and root dry weight (RDW) by 64.5% compared to the control plants (**Figures 2A–F**). Cereal crops like rice, maize, and wheat are considered glycophytes and exhibit high sensitivity to salt stress. In the current study, the reduced plant growth features can be attributed to saturating the cellular microenvironment with Na^+^ ions and the osmotic stress-induced ROS generation. Likewise, Zhang Y. et al. (2025) stated that high levels of Na^+^ inside cells interfere with vital ionic balances by competing with K^+^ and Ca^2+^ during enzymatic activities. This triggers a dual stress response involving osmotic imbalance due to reduced water availability and ionic toxicity resulting from Na^+^ buildup.

Contrarily, Fe_3_O_4_@Ce NCs alleviated these stress-induced effects in a dose-dependent manner and promoted the plant growth and biomass production under salt stress. The combined treatment of NaCl with 50 mg L^−1^ NCs resulted in the most significant improvement, with increases of 59.2% in SL, 79.4% in SFW, 52% in SDW, 72.8% in RL, 63.2% in RFW, and 76% in RDW compared to the NaCl-stressed plants alone (**Figures 2A–F**). Other treatments, including NaCl + 25 mg L^−1^ NCs and NaCl + 100 mg L^−1^ NCs, also showed a similar positive trend in improving growth parameters, though to a lesser extent. These improvements can be attributed to several factors complementary to a combined cerium-iron (Fe_3_O_4_@Ce) NCs, including an increase in photosynthesis and antioxidants, and the reduction of ROS and Na^+^ accumulation. Cerium oxide (CeO) NPs restrict excessive Na^+^ accumulation and retain leaf K^+^, maintaining a higher leaf K^+^/Na^+^ ratio (Liu et al., 2021), as well as enhancing α-amylase activity (Khan et al., 2021), and modifying the development of root apoplastic barriers (Rossi et al., 2017). Similarly, Iron oxide (FeO) NPs enhance the accumulation of organic osmolytes and antioxidants (Zhang Y. et al., 2025), increasing chlorophyll content, relative water content, and sugar levels under stress conditions (Moloudzadeh et al., 2024). Overall, NCs supplementation under salt stress effectively maintained plant growth, bringing it closer to that of healthy, non-stressed control plants.

### 3.3 Iron cerium NCs improved photosynthesis

**Figure 3:**
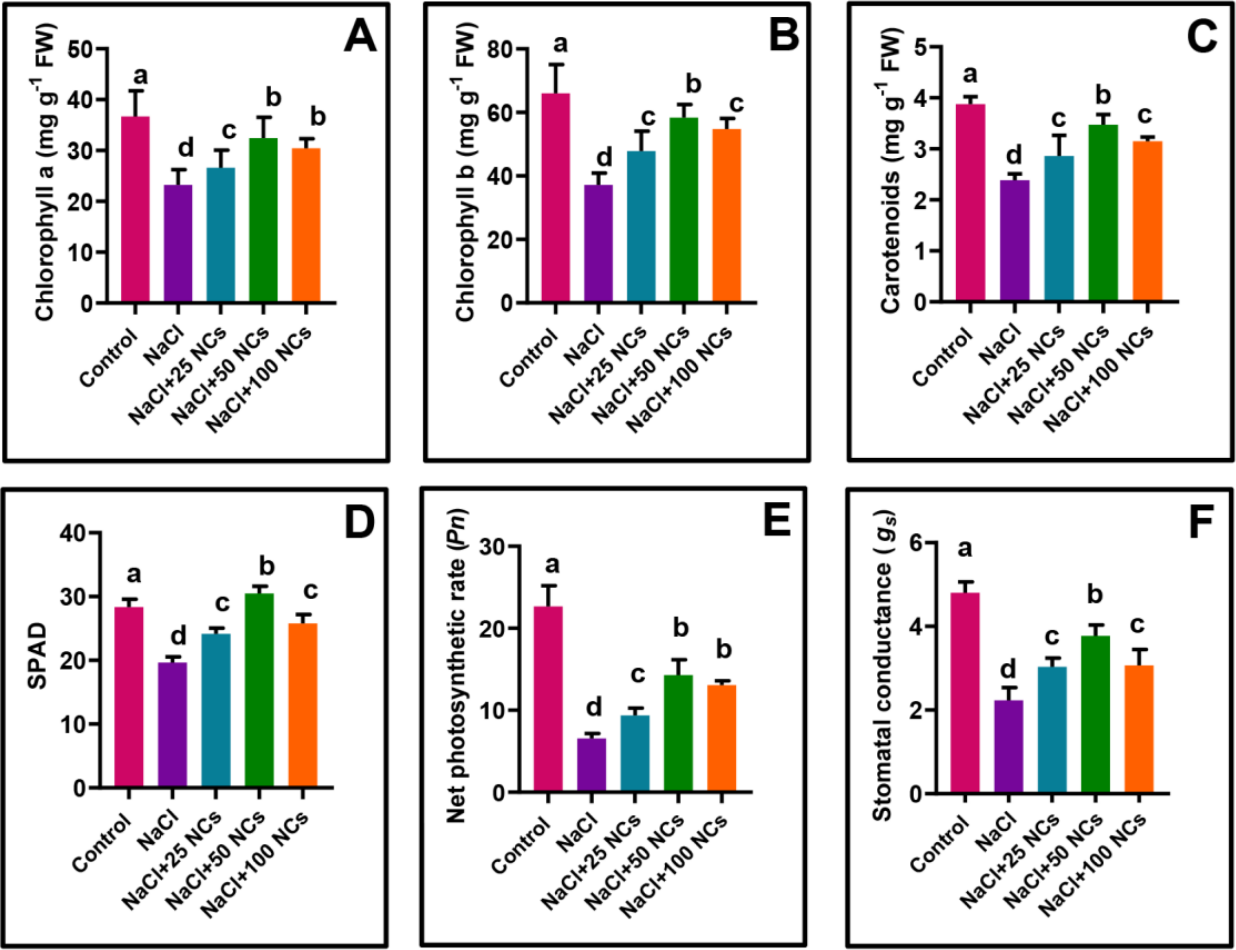
Effects of Fe_3_O_4_@Ce NCs on maize photosynthetic pigments and fluorescence under salt stress: **(A)** Chlorophyll a, **(B)** Chlorophyll b, **(C)** Carotenoids, **(D)** SPAD values, **(E)** Net photosynthetic rate, **(F)** Stomatal conductance at 25, 50, and 100 mg L^−1^ concentration.

Photosynthesis is essential for the survival of all living organisms, as it supports plant growth by producing the carbon backbone necessary for biomolecules. Our findings revealed that salt stress had a detrimental effect on the photosynthetic attributes of maize, substantially reducing chlorophyll a (36.6%), chlorophyll b (43.9%), carotenoids (38.5%), and SPAD values (31.3%). Chlorophyll constitutes a crucial pigment essential for facilitating photosynthesis in plants (Wu et al., 2017). Additionally, salt stress had an adverse effect on net photosynthetic rate and stomatal conductance (**Figures 3E, F**). Salt-induced reduction in photosynthesis is attributed to multiple factors, including disrupted enzymatic activity, inhibited chlorophyll synthesis, damage to the photosynthetic apparatus, impaired electron transport between PSII and PSI, and limited CO_2_ availability due to stomatal closure (Nadeem et al., 2020; Zahra et al., 2022). These factors collectively contribute to lower chlorophyll contents and consequently reduced photosynthesis.

The application of NCs improved the photosynthetic efficiency of the plants and alleviated the toxic effects of NaCl at concentrations of 25, 50, and 100 mg L^−1^. Among all treatments, 50 mg L^−1^ NCs under NaCl stress yielded the most pronounced improvement, as evidenced by significant increases in chlorophyll a (39.6%), chlorophyll b (57.2%), carotenoids (45.7%), and SPAD (55.1%). Similarly, this treatment also significantly boosted the net photosynthetic rate by 117.0% and stomatal conductance by 87.4% compared to NaCl-stressed plants (**Figures 3E, F**). The other treatments (NaCl + 25 mg L^−1^ NCs and NaCl + 100 mg L^−1^ NCs) also enhanced chlorophyll a, chlorophyll b, carotenoids, SPAD, net photosynthetic rate, and stomatal conductance relative to the salt-stressed control. However, NaCl + 50 mg L^−1^ NCs consistently outperformed the others in improving these traits. An increase in chlorophyll content ultimately results in absorbing more light energy and subsequently better photosynthesis (**Figures 3A–D**). Similar results have been reported for Fe NPs and CeO_2_ NPs under salt stress (Liu et al., 2021; Zhang Y. et al., 2025). Likewise, FeO NPs enhanced chloroplast development and accelerated the stabilization of PSII, as evidenced by improvements in chlorophyll (a + b), *Pn*, and Fv/Fm (Zhang Y. et al., 2025). Haydar et al. (2023) reported the role of Fe–Mn NC-doped graphene quantum dots in improving photosynthetic pigments, which enhances plant growth under salt stress. The involvement of CeO_2_ NPs in improving photosynthesis was also identified in Pea plants (Skiba et al., 2021). Studies reported that Na^+^ accumulation in the chloroplast reduced photosynthetic pigments, which ultimately impaired photosynthetic efficiency by lowering PSII quantum yield and electron transport rate (Yang et al., 2020; Yue et al., 2018). However, NCs reduced these abnormalities and collectively improved photosynthesis by reducing Na^+^ buildup and improving chlorophyll biosynthesis.

### 3.4 Iron cerium NCs improved the antioxidant system and reduced oxidative damage

**Figure 4:**
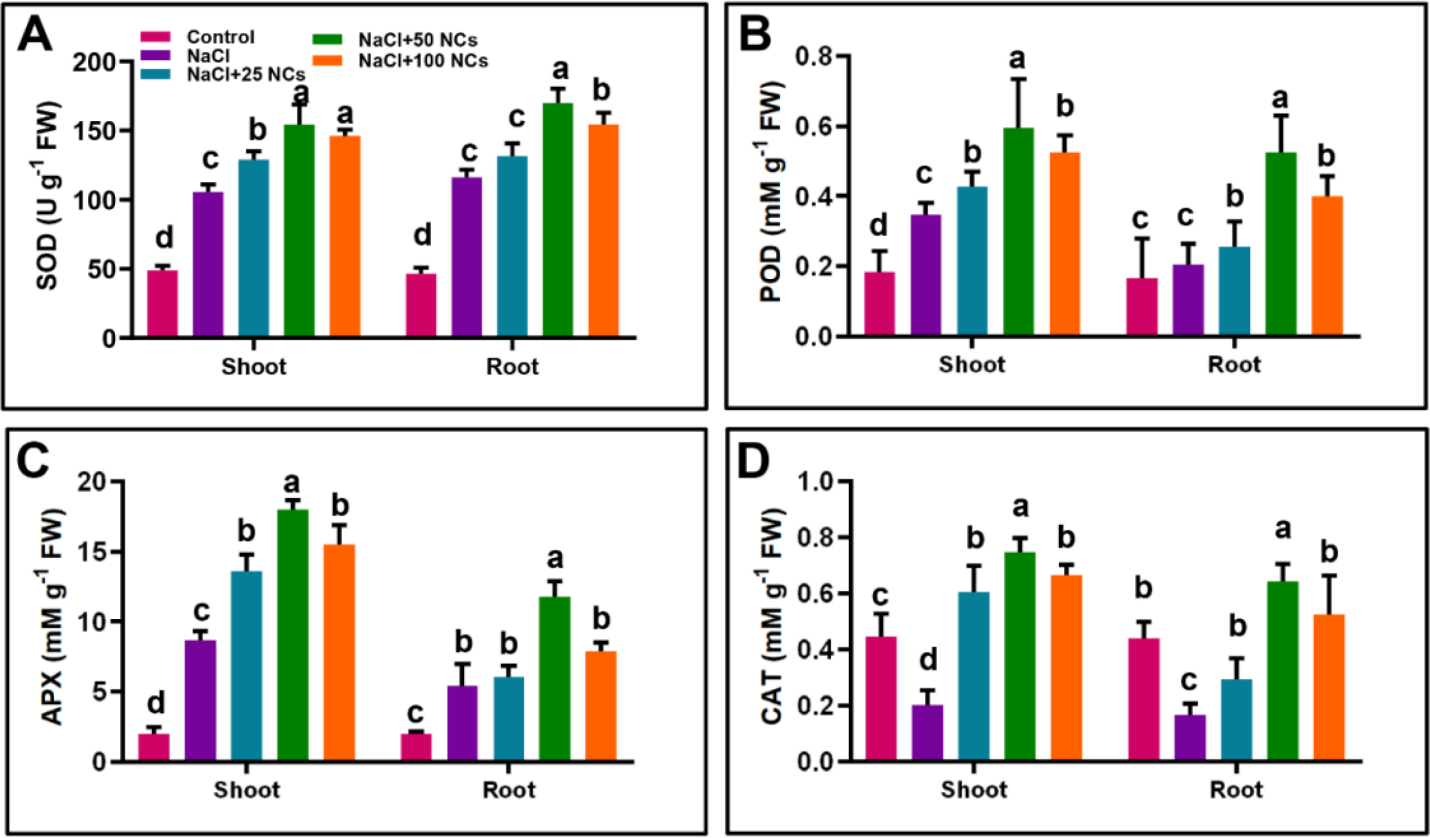
Effects of Fe_3_O_4_@Ce NCs on oxidative stress markers and antioxidant enzymes in maize under salt stress: **(A)** SOD, **(B)** POD, **(C)** APX, **(D)** CAT activities at 25, 50, and 100 mg L^−1^ concentration.

The overproduction of ROS induces oxidative damage, leading to impaired functions of proteins, lipids, and nucleic acids, and ultimately, cellular death (Ismail et al., 2022). In response to oxidative damage, plants’ defence relies on antioxidant enzymes such as SOD, POD, APX, and CAT to mitigate ROS-induced damage under stress conditions. Our results showed that salt stress significantly increased the activities of SOD, POD, and APX in both shoots and roots compared to the control plants (**Figures 4A–D**). Specifically, NaCl treatment led to an increase in SOD activity by 118% in shoots and 152% in roots, POD by 33.7% in shoots and 23.2% in roots, and APX by 354% in shoots and 177.1% in roots, respectively. In contrast, CAT activity was reduced by 54% in shoots and 61% in roots relative to the control. Interestingly, the application of Fe_3_O_4_@Ce NCs at different concentrations further enhanced the activities of these antioxidant enzymes, suggesting a stronger detoxification of ROS and improved oxidative stress tolerance in treated plants.

The combined treatment of NCs at 50 mg L^−^¹ under NaCl stress significantly enhanced SOD activity by 46.6% in shoots and 45.6% in roots compared to plants subjected to NaCl stress alone (**Figure 4A**). Similarly, this treatment markedly increased POD activity by 73.2% in shoots and 160.2% in roots relative to NaCl-only treated plants (**Figure 4B**). NCs at 50 mg L^−^¹ under NaCl stress also significantly increased APX activity by 107.4% in shoots and 116.4% in roots compared to the NaCl-only treatment (**Figure 4C**). Notably, salt stress alone reduced CAT activity in both shoots and roots compared to the other treatments. However, the application of NCs significantly enhanced CAT activity, with increases of 276.1% in shoots and 284.7% in roots relative to the control (**Figure 4D**).

Additionally, the relative expression of *GSH*, *SOD*, and *CAT* genes was largely consistent with the observed enzyme activities. Notably, *GSH* and *SOD* expression were statistically similar between the 25 and 50 mg L^−^¹ treatments, whereas *CAT* expression was higher at 50 mg L^−^¹. This correspondence between gene expression and enzyme activity suggests that NCs may regulate antioxidant defenses primarily at the transcriptional level by activating stress-responsive genes, while post-transcriptional processes such as enzyme synthesis, stability, and activation further contribute to the enhanced antioxidant capacity. The strongest response observed at 50 mg L^−^¹ likely reflects an optimal NPs concentration that efficiently stimulates defense signaling pathways without causing metabolic imbalance. At lower concentrations, the stimulus may be insufficient to fully activate antioxidant gene expression, whereas higher concentrations may reduce efficiency due to potential NPs aggregation or cellular stress. Both Fe and Ce likely contribute to this response by modulating redox signaling and triggering specific defense gene expression patterns, ultimately improving ROS detoxification and protecting plants from salt-induced oxidative damage. These findings are consistent with the results of Haghmadad Milani et al. (2024), who revealed that the application of CeO_2_ NPs boosted the antioxidant defense system in Spearmint (*Mentha spicata* L.) plants under salt stress.

**Figure 5:**
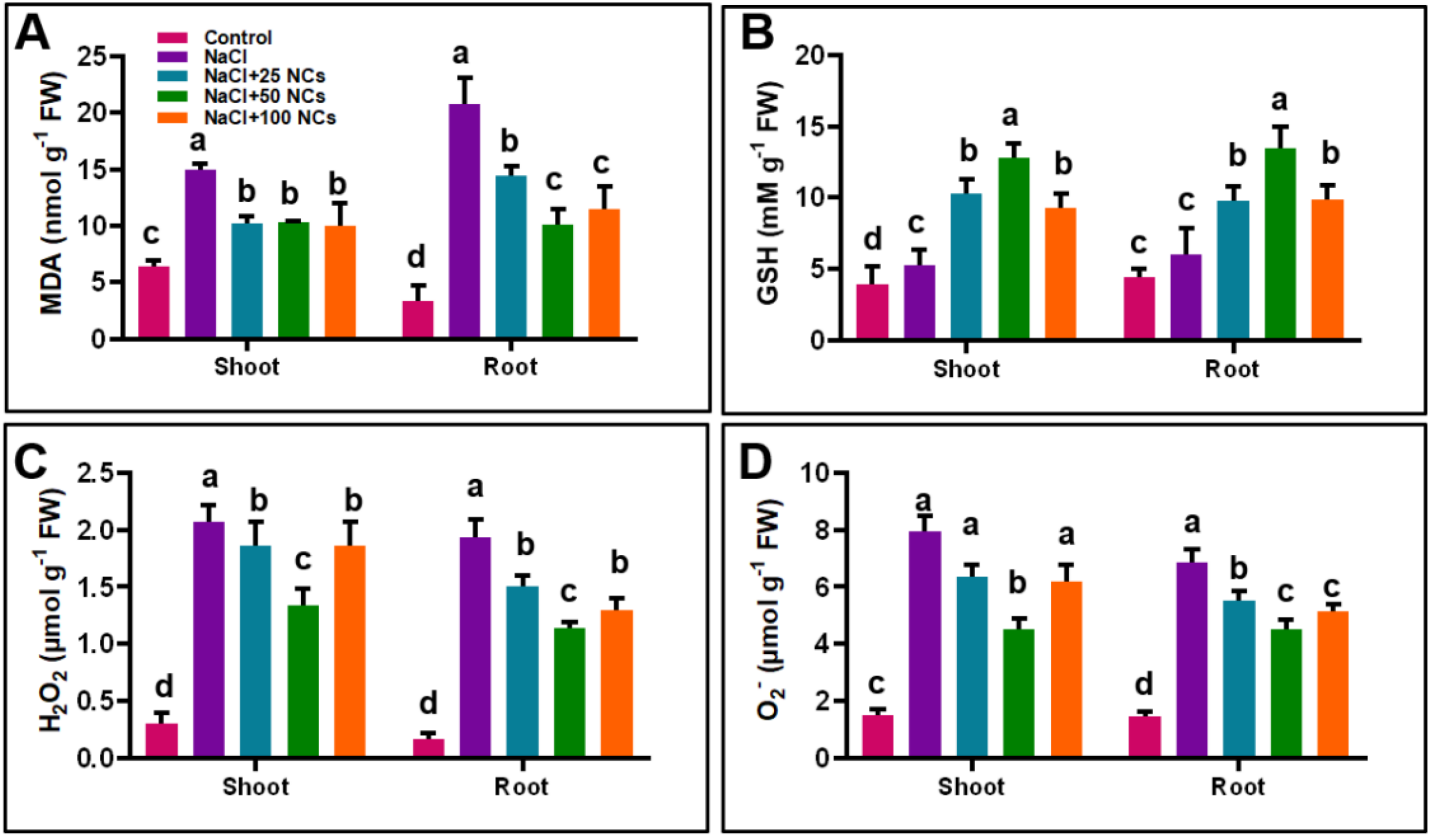
Effects of Fe_3_O_4_@Ce NCs on oxidative stress markers **(A)** MDA contents, **(B)** GSH activity, **(C)** H_2_O_2_ contents, and **(D)** O_2_^•^−^^ contents at 25, 50, and 100 mg L^−1^ concentration under salt stress.

The increase in antioxidant enzymes subsequently reduced the ROS and MDA accumulation in both roots and shoots (**Figure 5A**). Specifically, NC at 50 mg L^−1^ + NaCl treatment decreased H_2_O_2_ levels by 38.1% in the shoot and 27.46% in the root, and O_2_^•^−^^ levels by 43.4% in the shoot and 34.3% in the root, respectively. MDA levels in shoots were 30.7% for the NCs 25 mg L^−1^ + NaCl treatment, 51.4% for the 50 mg L^−1^ + NaCl treatment, and 44.9% for the 100 mg L^−1^ + NaCl treatment, relative to the NaCl-only group. These results suggest that NCs at 50 mg L^−1^ is the optimal concentration for mitigating lipid peroxidation and oxidative damage under salt stress, as indicated by substantial reduction in MDA levels. Similarly, the treatments NaCl+25 NCs, NaCl+50 NCs, and NaCl+100 NCs increased GSH activity by 95.25%, 142.12%, and 75.28% in the shoot, respectively, compared to the NaCl-only treatment. In the root, all the combined applications of NCs significantly increased the GSH activity in NaCl+25 NCs (63%), NaCl+50 NCs (124%), and NaCl+100 NCs (63.83%) as compared to NaCl alone (**Figure 5**).

### 3.5 Iron cerium NCs improved Ca^2+^, K^+^ and Mg^2+^ contents and reduced Na^+^ content

**Figure 6:**
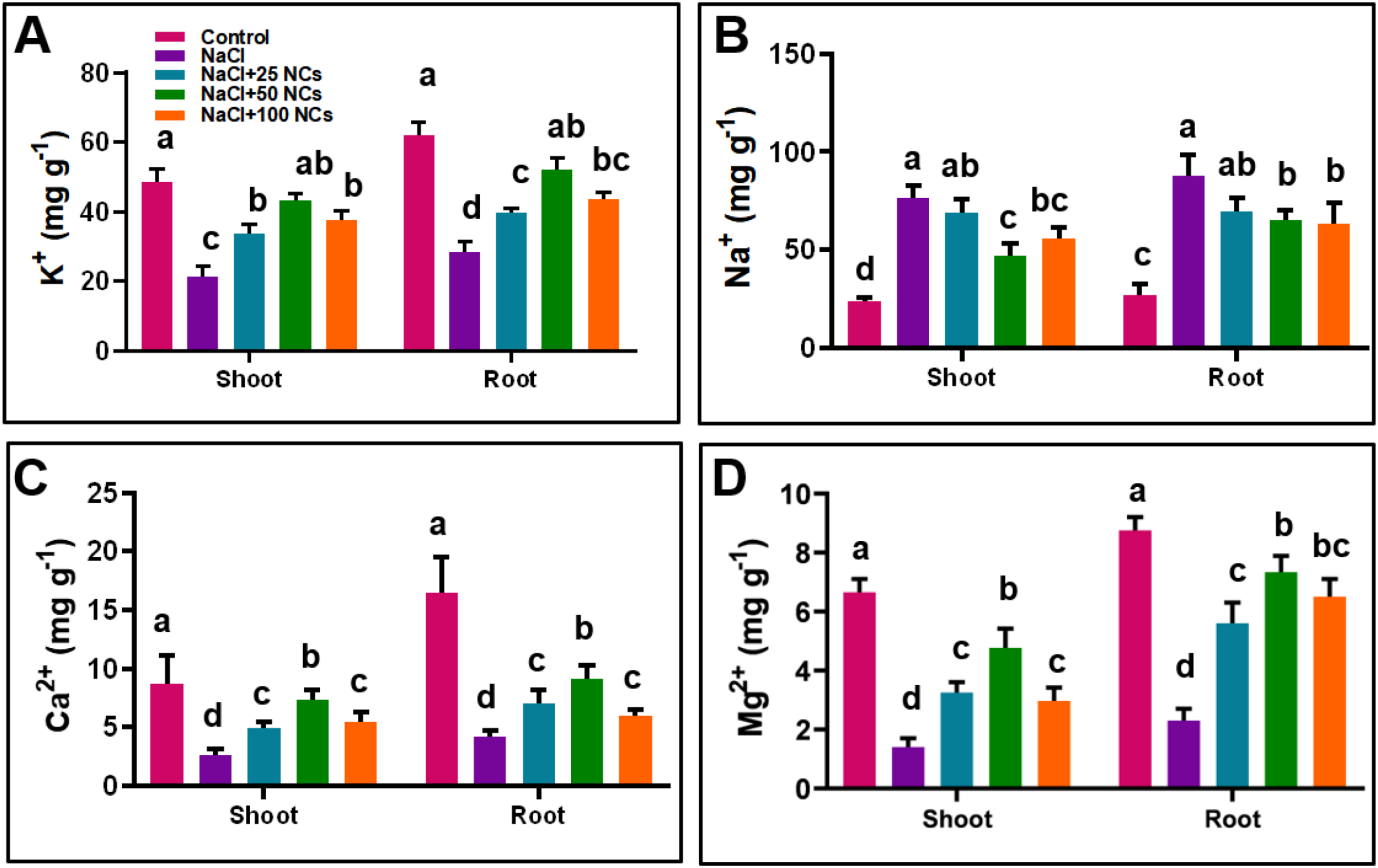
Effects of Fe_3_O_4_@Ce NCs on **(A)** Potassium, **(B)** Sodium, **(C)** Calcium, and **(D)** Magnesium contents at 25, 50, and 100 mg L^−1^ concentration under salt stress.

To determine the potential of NCs on endogenous Na^+^ content uptake, we measured the Na^+^ contents in shoots and roots of maize plants under different treatments (**Figure 6**). The results showed that salt stress alone significantly enhanced the Na^+^ content and reduced the key nutrients, such as K, Ca^2^**^+^**, and Mg^2^**^+^**, in both the roots and shoots (**Figures 6A, C, D**). In contrast, the foliar application of NCs with different concentrations effectively reduced the Na^+^ content in the presence of salt stress. Additionally, NCs at various concentrations alongside salt led to significant increases in K**^+^**, Ca^2^**^+^**, and Mg^2^**^+^** concentrations in both shoots and roots, while decreasing Na^+^ content compared to salt stress. The highest K^+^ levels were observed in the combined treatments of NaCl + 50 NCs (104.2%) for the plant’s shoot and (85.2%) root when compared to the NaCl alone. Similarly, use of NCs at 50 and 100 mg L^−1^ under NaCl resulted in a significant decrease (38.2%, 27.6%) and (26.4%, 28.2%) in Na^+^ content in both shoots and roots, respectively, when compared to NaCl alone (**Figure 6**). Compared to the control, Ca content in shoots increased by 88.2%, 176.2%, and 106.2% under the combined treatments of NaCl with 25, 50, and 100 mg L^−1^ NPs, respectively. Interestingly, a similar trend was observed for Mg^2^**^+^** content in both shoots and roots, with significant improvements seen across all treatments (NaCl+25NCs, 50NCs, and 100NCs). The Mg^2^**^+^** content was significantly higher under the combined treatments of NaCl with 50 mg L^−1^ NCs (240% in shoots and 217.2% in roots) and NaCl with 100 mg L^−1^ NCs (92.8% in shoots and 182.5% in roots), compared to salt stress alone. These findings are in accordance with Anjum et al. (2025) who reported that NPs can enhance the essential nutrient profile of plants under salt stress by reducing the uptake of Na^+^ ions.

### 3.6 Iron cerium NCs enhanced osmolytes and total protein

The results showed that salt stress significantly increased proline content compared with the control, and foliar application of NCs further enhanced this accumulation. The highest proline level was observed at 50 mg L^−1^ NCs, which was significantly greater than both the control and salt-stressed plants (101% and 46% respectively). However, 25 and 100 mg L^−1^ showed no significant difference from each other, with increases of 17% and 14%, respectively, as compared to stressed plants. A similar trend was observed for glycine betaine. Its content increased under stress conditions as part of the plant’s coping mechanism and was further enhanced by NC application, with the most substantial effect at 50 mg L^−1^, resulting in 241% and 85% increases compared to the control and salt stress, respectively. In contrast, salt stress caused a sharp reduction in soluble sugars and total proteins (by 62.4% and 56.5%, respectively (**Supplementary Figure 3**). NC treatment markedly alleviated these declines, increasing soluble sugars by 110% and proteins by 94.1% compared with salt stress alone. The most pronounced recovery occurred with 50 mg L^−1^ NCs, while no significant difference was observed between 25 mg L^−1^ and 100 mg L^−1^.

Osmolytes such as proline, glycine betaine, and soluble sugars accumulate in plants to protect them against osmotic stress induced by various environmental stressors (Wu, 2018). Our results suggest that NCs help maintain cellular osmotic balance under salt stress, likely by promoting ion exclusion or compartmentalization and enhancing the accumulation of compatible solutes such as proline and glycine betaine. Zhu et al. (2022) showed that glycine betaine improves the growth of maize under salt stress by regulating cellular Na^+^ homeostasis, enhancing Na^+^ efflux from roots, reducing the Na^+^/K^+^ ratio, and upregulating Na^+^/H^+^ antiporter and H^+^-H-ATPase genes. Rhaman et al. (2024) applied proline and glycine betaine exogenously under salt stress and reported that their supplementation enhanced seed germination and seedling growth traits while mitigating pigment degradation. Moreover, proline and glycine betaine reduced the accumulation of MDA and ROS, thereby alleviating oxidative stress in salt-stressed maize plants and increasing the activity of enzymatic antioxidants. Similarly, proline is widely recognized as a reliable stress marker in various plant species (Liang et al., 2013). It plays a key role in mitigating salinity damage by acting as an osmoprotectant that maintains cell turgor and protects cellular structures from water deficit and high salt conditions. Zhu et al. (2022) reported that glycine betaine decreased the accumulation of Na^+^ in both shoots and roots, primarily due to a reduced rate of Na^+^ uptake in roots and enhanced Na^+^ efflux from leaf cells.

### 3.7 Iron cerium NCs regulated the stomatal structure and improved their number

**Figure 7:**
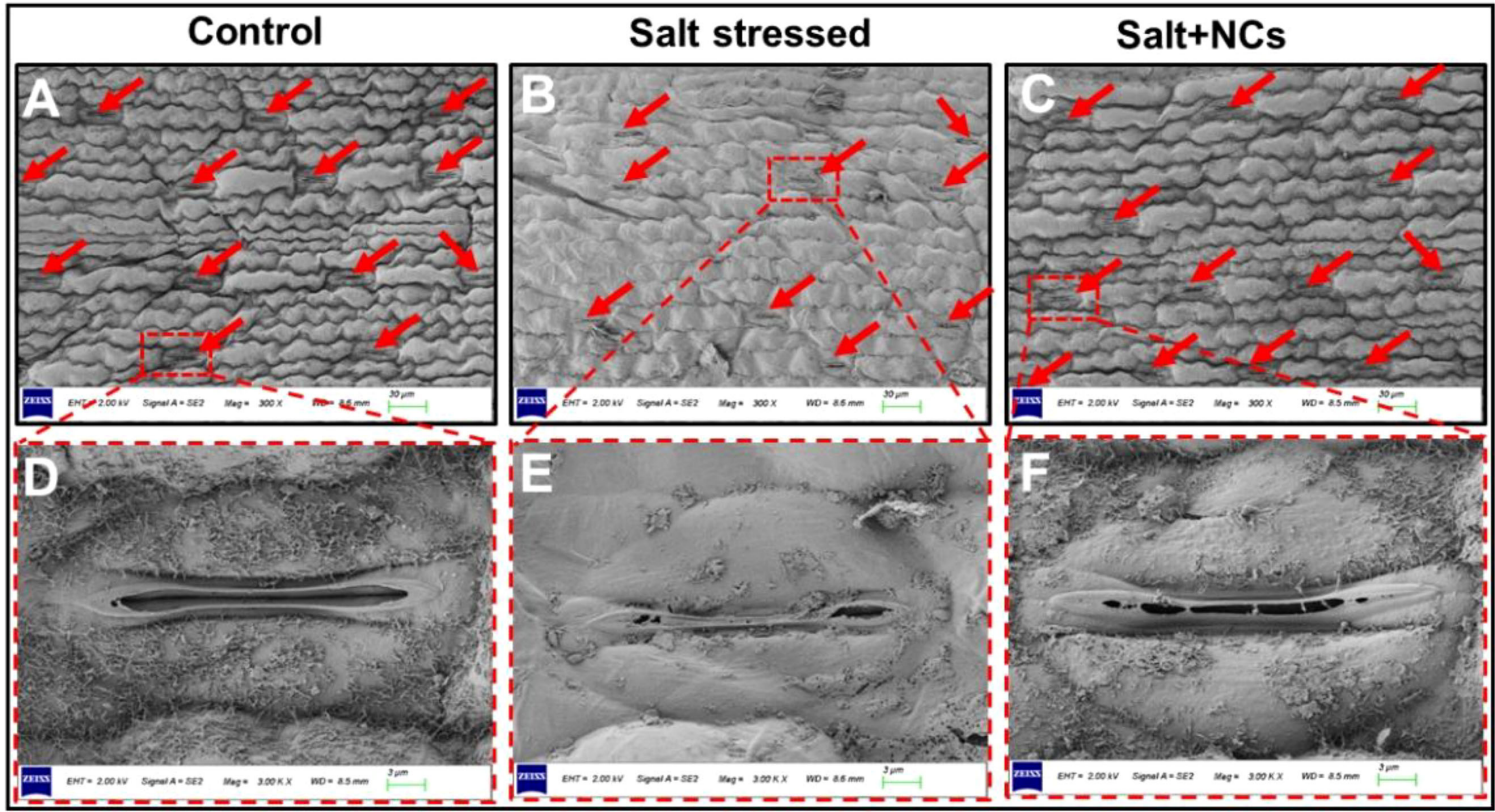
Effects of Fe_3_O_4_@Ce NC on stomatal characteristics of maize under salt stress: **(A–C)** number of stomata, **(D–F)** Microscopic observations of stomata and guard cells at 50 mg L^−1^ Fe_3_O_4_@Ce NCs concentration. (Scalebar: 30 µm **(A, B, C)**, and 3 µm **(D, E, F)**.

Stomata play a crucial role in regulating gas exchange and respiration in plants, thereby contributing significantly to plant survival under stressful conditions. In this study, salt stress caused noticeable damage, including distorted guard cells, reduced stomatal density, and predominantly closed stomata, likely due to partial or complete plasmolysis of guard cells (**Figure 7**). The change in stomatal aperture may be attributed to enhanced levels of ROS and MDA content (**Figure 5**). The excessive level of salt stress reduced photosynthesis by disrupting stomatal conductance and the thylakoid membrane (Javaid et al., 2024). High Na^+^ and Cl^−^ accumulation in leaves disrupts cellular ion homeostasis and interferes with K^+^ fluxes in guard cells, impairing stomatal opening and closing (Zuo et al., 2025).

In contrast, the NC application alleviated salt-induced damage, likely by maintaining osmotic balance and preserving water retention in guard cells, thus supporting stomatal opening. The uptake of K^+^ ions raises guard cell turgor pressure, facilitating stomatal opening (Kashtoh and Baek, 2021). Recently, Li Y. et al. (2025) reported that NPs mitigate the adverse effects of drought stress on stomatal aperture, resulting in enhanced photosynthetic performance in cotton plants. These outcomes demonstrate that the foliar application of NCs may reduce salt-induced ROS accumulation, resulting in sustained guard cell turgor pressure and subsequent stomatal opening. This suggests that NCs play a protective role in maintaining stomatal function under salinity stress.

### 3.8 Iron cerium NCs improved mesophyll and root ultrastructure

**Figure 8:**
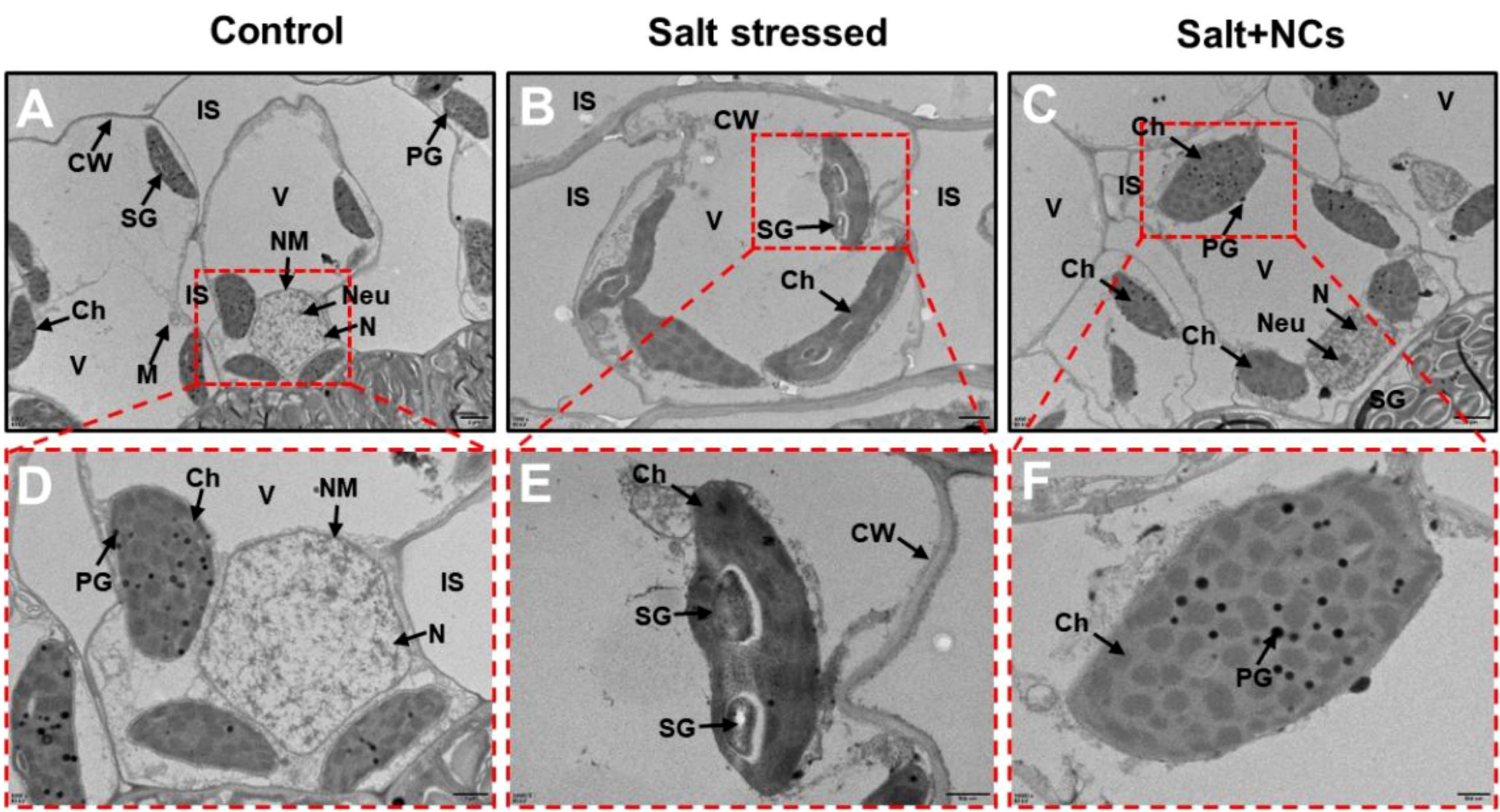
Effects of Fe_3_O_4_@Ce NCs on mesophyll cell ultrastructure of maize under salt stress. **(A–C)** general overview of the organelle and **(D-F)** nucleus, chloroplast observation under control, stress, and NCs (50 mg L^−1^ Fe_3_O_4_@Ce) + stress conditions. Abbreviations: IS, intercellular spaces; M, mitochondrion; V, vacuole; CW, cell wall; Ch, chloroplast; N, nucleus; Neu, nucleolus; NM, nuclear membrane; SG, starch granule; PG, plastoglobuli.

To further support our findings, TEM was used to examine the ultrastructure of the leaf mesophyll and root apical cells. Salt-stressed seedlings showed severe cellular damage in both shoot and root cells (**Figure 8**). In mesophyll cells, it caused disrupted and irregular nuclei, deformed chloroplasts with disorganized or dissolved thylakoids, swollen mitochondria with disturbed cristae, reduced starch granules, and cracked or broken cell walls. Additionally, salt-stressed roots exhibited severe cellular damage, characterized by numerous small vacuoles within the cytoplasm, poorly defined nuclear membranes, and disintegrated nucleoli, indicating disruption of the cellular matrix. Previously, salt stress has been found to induce toxic effects on the cellular ultrastructure of various plant species (Liu et al., 2025; Wu et al., 2024). Javaid et al. (2024) reported that elevated levels of NaCl stress induced structural impairment to root tips and leaf mesophyll cells, primarily due to the excessive production of ROS, leading to oxidative damage at the cellular level.

**Figure 9:**
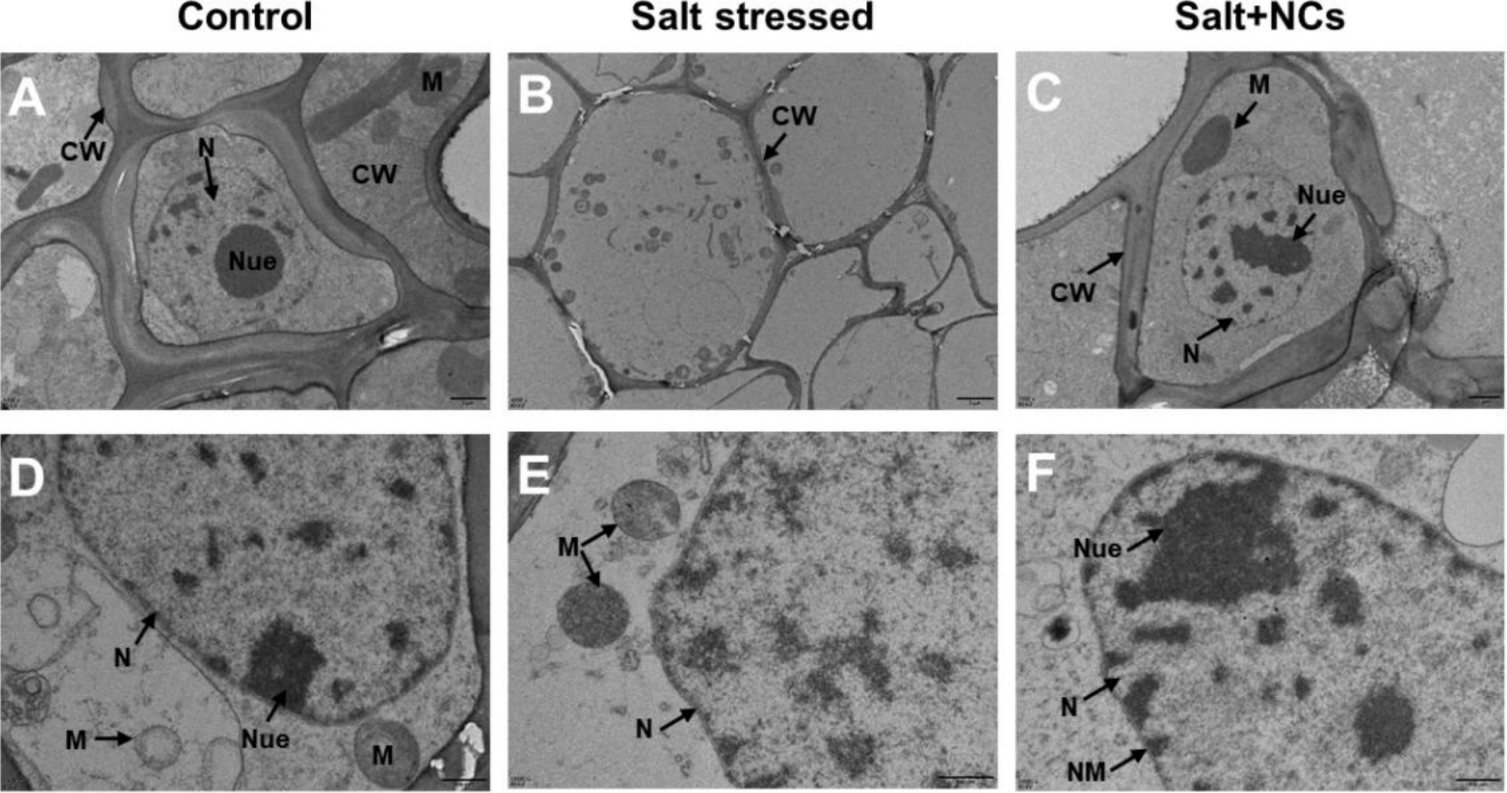
Effects of Fe_3_O_4_@Ce NCs on root ultrastructure of maize under salt stress via TEM. Abbreviations: M, mitochondrion; CW, cell wall; N, nucleus; Neu, nucleolus; NM, nuclear membrane.

Interestingly, the foliar application of NCs reversed the salt-induced toxic effects on maize leaf mesophyll cells (**Figure 8**). The positive potential of NCs in the protection of cellular ultrastructure is also linked with the role of NCs in the reduction of stress indicators (H_2_O_2_, O_2_^•–^ and MDA contents), likely to maintain the cellular integrity by reducing the lipid membrane peroxidation under salt stress (**Figure 5**). The positive potential of NPs to protect the cellular ultrastructure of plants under salt stress has been previously reported (Kareem et al., 2025). The ultrastructural changes in the root tips of maize seedlings under different treatments are shown in **Figure 9**. The foliar application of NCs reduced the salt-induced damage to the cellular ultrastructure of maize roots (**Figure 9C**). A potential mechanism is that NCs diminished the salt-induced ROS accumulation in the cellular compartments and mitigated cellular damage by stabilizing membrane integrity.

## 4 Conclusion

In this study, we synthesized cerium-coated triiron tetraoxide NCs via the co-precipitation method and evaluated their effectiveness in mitigating salt stress in maize. Salt stress induces osmotic imbalance, disturbs ionic homeostasis, and results in ion toxicity. These disruptions collectively suppress plant growth, reduce biomass and photosynthetic efficiency, and damage the ultrastructure of mesophyll cells and root tips. Application of NCs mitigated these adverse effects by enhancing antioxidant enzyme activities and lowering ROS accumulation. Additionally, NCs helped alleviate ion toxicity by restricting Na^+^ uptake, promoting intracellular Na^+^ sequestration, and improving cytosolic K^+^ levels. These findings highlight the potential of iron-based NCs as biocompatible and efficient agents for enhancing plant resilience to salinity. Foliar application at 50 mg L^−1^ proved optimal, resulting in significant improvement in growth attributes and the least cellular damage. This research offers valuable insights into innovative strategies for enhancing crop resilience and productivity in saline soils, thereby contributing to sustainable agricultural practices. Field-based studies are necessary to validate these results across diverse environments and to gain a deeper understanding of their performance under practical agricultural conditions. Future studies should aim to elucidate the underlying molecular mechanisms, optimize application protocols, and assess the long-term impacts and broader applicability of these NCs across diverse agricultural systems.

## Data Availability

The original contributions presented in the study are included in the article/**Supplementary Material**. Further inquiries can be directed to the corresponding authors.
