# Supplementary material for: Cerium-coated Fe3O4 nanocomposite enhances salt stress tolerance in maize by modulating photosynthetic efficiency, antioxidant defense, and cellular ultrastructure
Source: Front Plant Sci. 2026 Apr 15;17:1768765. doi: 10.3389/fpls.2026.1768765 (PMC13124587; doi:10.3389/fpls.2026.1768765)
Supplement: Supplementary file 1 [file SupplementaryFile1.docx]

Supplementary Material


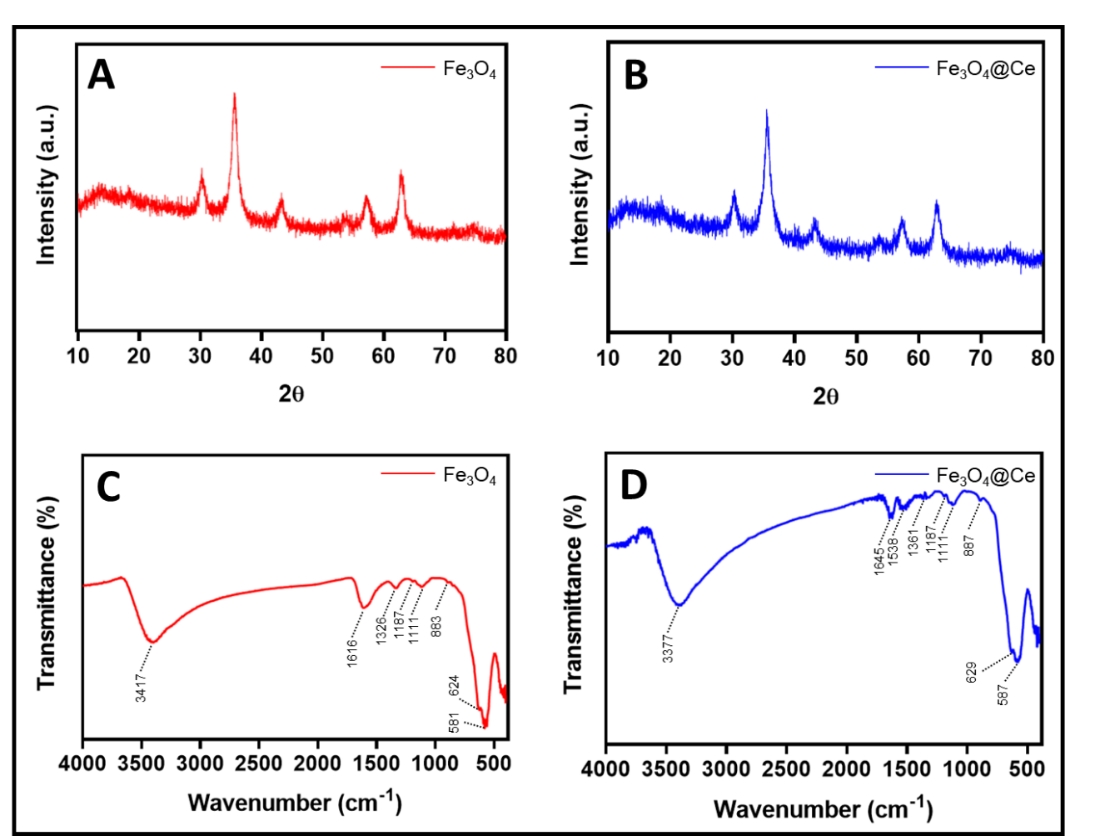


Figure S1. (A) XRD pattern of Fe_3_O_4_ NPs and (B) Fe_3_O_4_@Ce, (C) FTIR spectra of Fe_3_O_4_ NPs and (D) the Fe_3_O_4_@Ce nanocomposite, showing noticeable shifts in absorption peaks, which indicate successful cerium coating on the NPs surface.


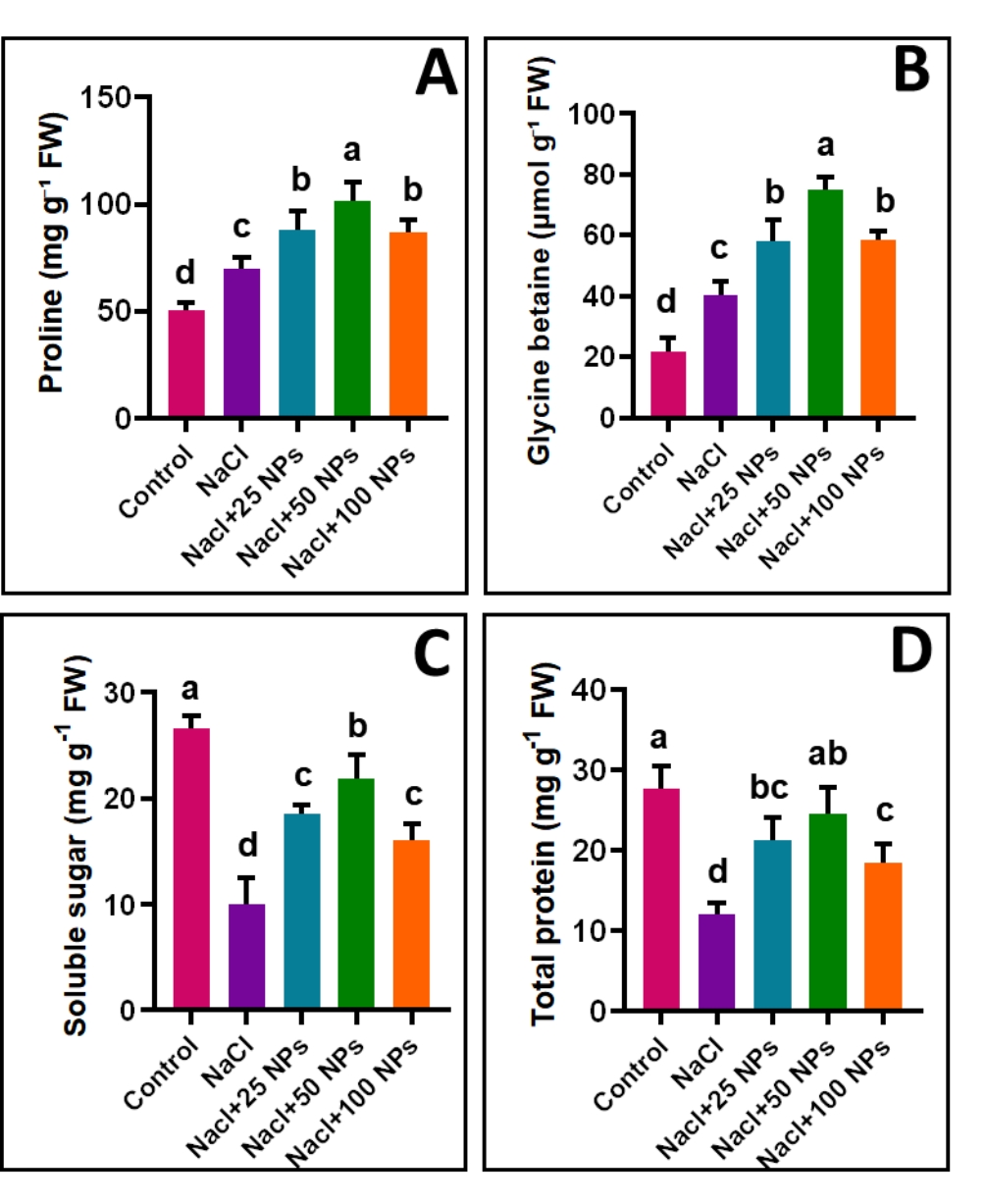


Figure S2. Effects of Fe_3_O_4_@Ce NCs on osmolytes and total soluble sugar, total protein under salt stress: (A) proline contents, (B) glycine betaine, (C) soluble sugar, (D) total protein at 25, 50, 100 mg L⁻^1^ concentration under salt stress.


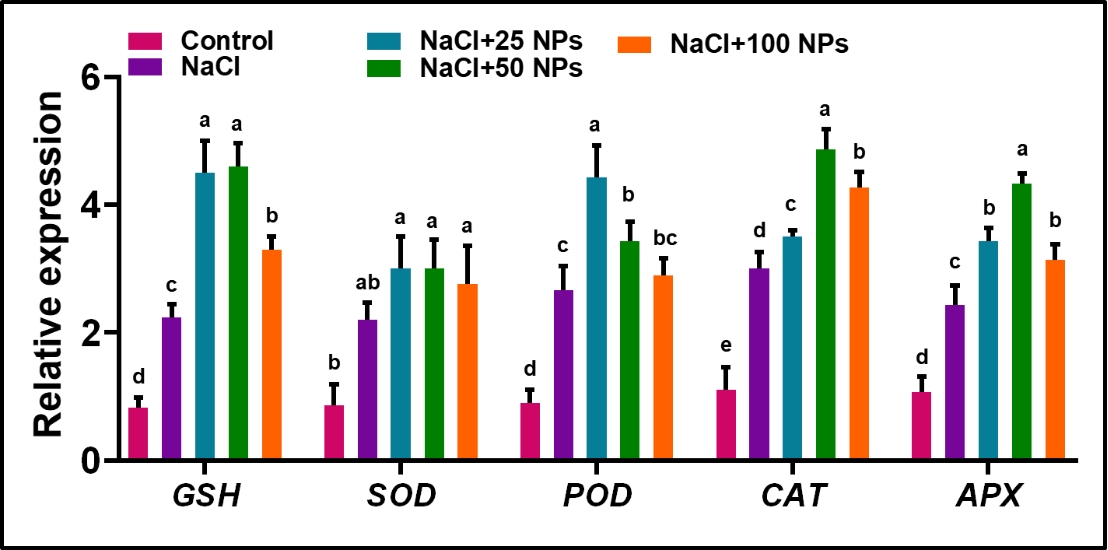


Figure S3. Effects of different concentrations of nanocomposite (25, 50, 100 mg L⁻^1^) on the transcript level of the antioxidant genes in the leaves of maize under salt stress.

Table S1. Primers used for qRT-PCR analysis in the present study.

| Gene name | Forward primer | Reverse primer |
| --- | --- | --- |
| *Actin* | GATGATGCGCCAAGAGCTG | GCCTCATCACCTACGTAGGCAT |
| *ZmGSH* | GGGTTGTGGAGCTTGGATAA | GTCCTGCAAATCCTGACCAT |
| *ZmSOD* | TTCGCCGCTCCCTATTCC | GTCCTGTCGATATGCACCCA |
| *ZmPOD* | ATGAGGTCCACTTGGTTGGC | CAGTCGTGGAAGTGCATCCG |
| *ZmCAT* | GCAAGGAGAACAACTTCAAGC | AGACCAGTTGGAGAGCCAGA |
| *ZmAPX* | GGGACTACGCGGAGTCACAC | GTTGTTGGATTCGCACAGGT |
